# Growth of single-crystalline Bi_2_Te_3_ hexagonal nanoplates with and without single nanopores during temperature-controlled solvothermal synthesis

**DOI:** 10.1038/s41598-019-47356-5

**Published:** 2019-07-25

**Authors:** Yuichi Hosokawa, Koji Tomita, Masayuki Takashiri

**Affiliations:** 1 Department of Materials Science, Tokai University, 4-1-1 Kitakaname, Hiratsuka, Kanagawa 259-1292 Japan; 2 Department of Chemistry, Tokai University, 4-1-1 Kitakaname, Hiratsuka, Kanagawa 259-1292 Japan

**Keywords:** Nanoparticles, Nanoparticles

## Abstract

Bismuth telluride (Bi_2_Te_3_) is a promising thermoelectric material for applications near room temperature. To increase the thermoelectric performance of this material, its dimensions and thermal transport should be decreased. Two-dimensional nanoplates with nanopores are an ideal structure because thermal transport is disrupted by nanopores. We prepared Bi_2_Te_3_ nanoplates with single nanopores by a solvothermal synthesis and investigated their structural and crystallographic properties. The nanoplates synthesized at a lower reaction temperature (190 °C) developed single nanopores (approximately 20 nm in diameter), whereas the nanoplates synthesized at a higher reaction temperature (200 °C) did not have nanopores. A crystal growth mechanism is proposed based on the experimental observations.

## Introduction

**Figure 1:**
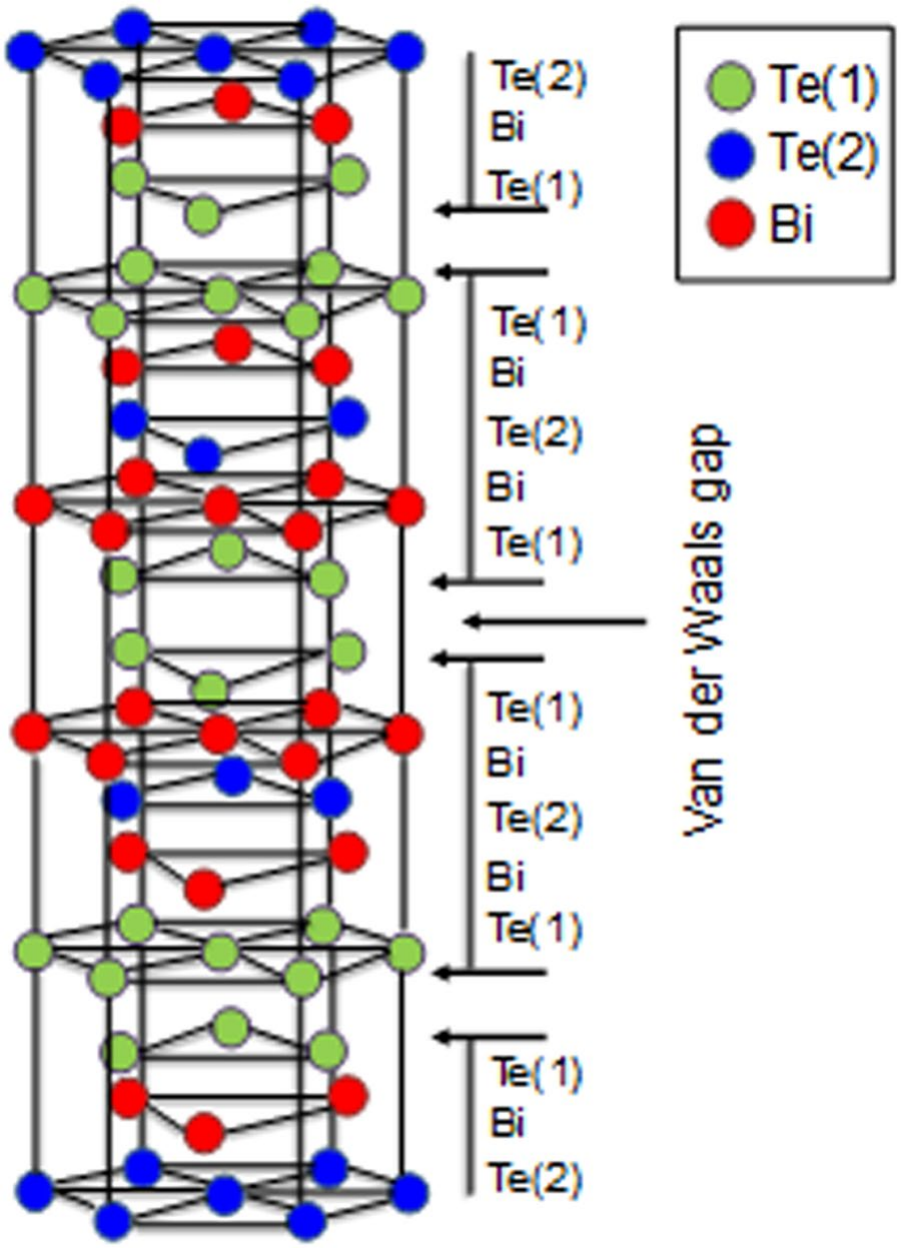
Schematic crystal structure of Bi_2_Te_3_.

Bismuth telluride (Bi_2_Te_3_) has attracted intense attention as a functional material in applications such as traditional thermoelectric systems^1,2^, three-dimensional (3D) topological insulators^3,4^, and photovoltaic materials^5^. The crystal structure of Bi_2_Te_3_ is a rhombohedral tetradymite-type with the space group R-3m, and is described by a hexagonal unit cell, as shown in Fig. 1. The unit cell is composed of five covalently bonded monatomic sheets along the *c*-axis in the sequence –Te(1)–Bi–Te(2)–Bi–Te(1)–Te(1)–Bi–Te(2)–Bi–Te(1)–. Here, (1) and (2) denote two different chemical states of the anions. The nature of the bonding between neighboring Te(1) layers is a weak van der Waals attraction, whereas the bonding between bismuth and tellurium atoms is mainly covalent with a small ionic contribution. Because of this crystal structure, the lattice constant of the *c*-axis (3.045 nm) is approximately seven times as large as that of the *a*, *b*-axis (0.438 nm), leading to remarkable anisotropic behavior^6–8^.

Recently, nanosized Bi_2_Te_3_ materials have been shown to exhibit excellent thermoelectric performance^9^. This high performance is mainly attributed to the effects of the low dimensionality and the reduction of the lattice thermal conductivity at phonon scattering centers^10–12^. Among various shapes of nanosized Bi_2_Te_3_ materials, including nanoplates^13–15^, nanoparticles^16–18^, and nanocrystalline films^19–22^, nanoplates are the most appropriate shape to take advantage of the inherent features of the Bi_2_Te_3_ crystal structure. This is because Bi_2_Te_3_ nanoplates are single-crystalline and have a hexagonal shape similar to that of the unit cell. To prepare Bi_2_Te_3_ nanoplates, solvothermal and hydrothermal syntheses are the most practical methods because of their cost efficiency and simplicity. In these syntheses, a precursor solution is placed in a pressure vessel and heated to temperatures much higher than their normal boiling points to enhance the chemical reaction and yield nanoplates. The difference between the solvothermal and hydrothermal syntheses is whether the precursor solution is non-aqueous (solvo) or aqueous (hydro).

Bi_2_Te_3_ nanoplates with a hexagonal shape are generally synthesized by solvothermal methods^23,24^. Typical Bi_2_Te_3_ nanoplates take the shape of a regular hexagon and have atomically flat surfaces^25,26^. Thermoelectric performance can be increased by forming optimized nanopores in the material, which decrease the thermal conductivity while preserving the electrical properties^27^. Therefore, if nanopores are formed in nanoplates, the thermoelectric performance of the nanoplates might be expected to further increase. However, to the best of our knowledge, there have been no reports on the crystal growth of Bi_2_Te_3_ nanoplates containing nanopores.

In this study, we prepare Bi_2_Te_3_ hexagonal nanoplates with single nanopores based on a solvothermal synthesis. For comparison, Bi_2_Te_3_ hexagonal nanoplates with no nanopores were also prepared using solvothermal synthesis by changing the reaction temperature. The structural and crystallographic characteristics of the nanoplates were investigated. On the basis of our experimental observations and understanding of the crystal growth, the formation of the nanoplates with nanopores is discussed.

## Methods

Single-crystalline Bi_2_Te_3_ hexagonal nanoplates were formed in a solvothermal synthesis. The basic experimental method has been described in our previous work^28,29^. The nanoplates were prepared in a Teflon-container within a stainless-steel autoclave, involving a reaction under high pressure and high temperature. Stirring was applied with a hot plate and magnetic stirrer. Bi_2_O_3_ (Fujifilm Wako Pure Chemical Co., >99.9%) and TeO_2_ (Kojundo Chemical Laboratory Co., Ltd., >99.9%) were used as Bi_2_Te_3_ sources, and C_2_H_6_O_2_ (Fujifilm Wako Pure Chemical Co., >90.0%) was used as a ligand. NaOH (Fujifilm Wako Pure Chemical Co., >97.0%) and polyvinyl pyrrolidone (Fujifilm Wako Pure Chemical Co., K30, M_s_ ~40,000) were contained in the solution. All chemicals were used as received without further purification. The Bi_2_Te_3_ nanoplates were synthesized according to the following procedure: 0.4 g of PVP was dissolved in ethylene glycol (18 mL), followed by the addition of Bi_2_O_3_ (0.02 mol/L), TeO_2_ (0.07 mol/L), and 2 mL of a NaOH solution (5.0 mol/L). The resulting precursor solution was sealed in the autoclave and heated and maintained at either 190 °C or 200 °C for 4 h with magnetic stirring at 500 rpm. After the synthesis, the products were allowed to cool down below 50 °C naturally. The residue was washed several times with distilled water and absolute ethanol and the products were collected by centrifugation. Finally, the products were dried under vacuum at 60 °C for 24 h.

We analyzed the microstructure of Bi_2_Te_3_ nanoplates with a field emission scanning electron microscope (FE-SEM, Hitachi S-4800). The precise structure of the nanoplates was analyzed with a high-resolution transmission electron microscope (TEM, JEOL JEM-ARM200F) and selected area electron diffraction (SAED) at an accelerating voltage of 200 kV. The chemical composition of the nanoplates was determined with an electron probe microanalyzer (EPMA, Shimadzu EPMA-1610). The compositions of the samples were calibrated using the ZAF4 program installed with the EPMA-1610. X-ray diffraction (XRD, Rigaku MiniFlex600) analyses were performed to determine the crystal lattice structure and the phase purity of the nanoplates. We used Cu-Kα radiation at a scanning rate of 0.02°/s over the 2θ range of 10° to 80°.

## Results and Discussion

**Figure 2:**
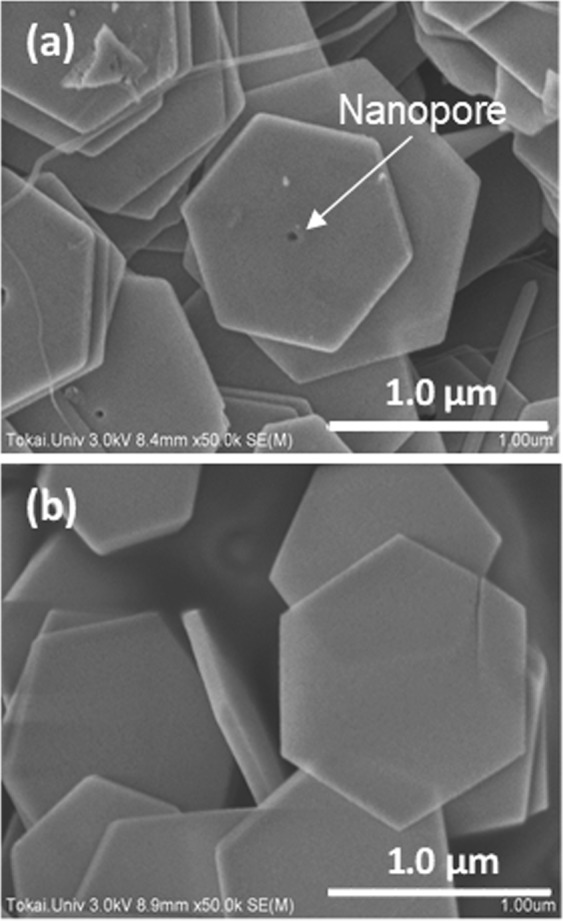
SEM images of the Bi_2_Te_3_ nanoplates synthesized at (**a**) 190 °C and (**b**) 200 °C.

Figure 2 shows SEM images of the Bi_2_Te_3_ nanoplates formed at different reaction temperatures in the solvothermal synthesis. At a reaction temperature of 190 °C (Fig. 2(a)), we obtained hexagonal nanoplates with edge lengths of approximately 1 μm. A single nanopore appeared in the center of the nanoplates. We also observed several samples manufactured under the same conditions as the sample shown in the SEM image and found a nanopore in the center of almost every nanoplate (The detailed information is provided in Fig. S1). Hence, the nanopores were naturally generated in the crystal growth process and not caused by accidental collisions between the nanoplates. At a reaction temperature of 200 °C (Fig. 2(b)), the nanoplates had a similar edge length of approximately 1 μm but no nanopores were observed. We also fabricated Bi_2_Te_3_ nanoplates at reaction temperatures of 180 °C and 230 °C, and observed their surface morphologies using FE-SEM (The detailed information is provided in Fig. S2). As a result, single nanopores were obtained in the nanoplates at 180 °C, but not in those formed at 230 °C. Therefore, we conclude that the synthesis at relatively low reaction temperatures yielded nanoplates with single nanopores and the nanopores disappeared as the reaction temperature was increased.

**Figure 3:**
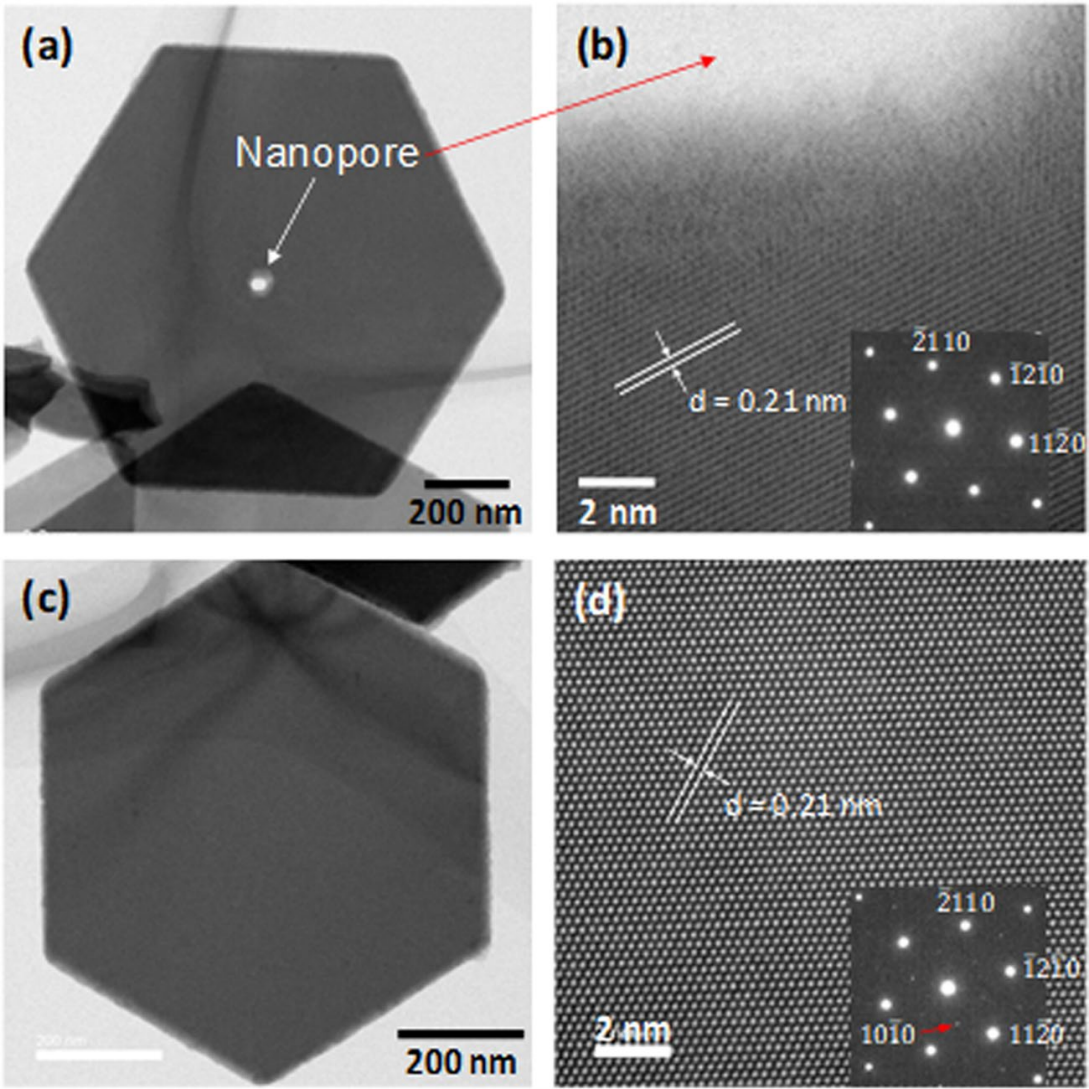
(**a**) TEM image of Bi_2_Te_3_ nanoplate with a single nanopore synthesized at 190 °C. (**b**) Corresponding SAED pattern and HRTEM image of the nanoplate in (**a**). (**c**) TEM image of Bi_2_Te_3_ nanoplate synthesized at 200 °C. (**d**) Corresponding SAED pattern and HRTEM image of the nanoplate in (**c**).

To further investigate the precise structure of the Bi_2_Te_3_ nanoplates, we performed TEM observations, as shown in Fig. 3. The TEM images of the Bi_2_Te_3_ nanoplates synthesized at a reaction temperature of 190 °C are shown in Fig. 3(a). The nanoplates had sharp edges with a distance of approximately 1 μm between the opposite edges, and a very smooth surface, which indicated excellent crystallinity. The mesh structure behind the nanoplate was visible indicating the low thickness of the plate. The size of the nanopores was approximately 20 nm, and each was located close to the center of each nanoplate. Figure 3(b) shows high-resolution TEM (HRTEM) images of the nanoplates near the nanopores. The SAED pattern inset in Fig. 3(b) shows a hexagonal symmetry diffraction spot pattern, which indicates its single-crystalline nature. Lattice fringes cannot be seen in the range of approximately 5 nm from the nanopore, suggesting an amorphous structure in this region. However, clear lattice fringes were observed in this region at distances greater than 5 nm from the edges of the nanopores. The lattice fringes were structurally uniform with a spacing of 0.21 nm, which is in good agreement with the d values of the (110) planes of rhombohedral Bi_2_Te_3_. From Fig. 3(a,b), we conclude that single-crystal nanoplates grew along the (00*l*) planes except for regions near the nanopores. Figure 3(c) shows a TEM image of a Bi_2_Te_3_ nanoplate synthesized at a reaction temperature of 200 °C. The shape of the nanoplate was almost the same as that synthesized at 190 °C except for the presence of the nanopore. The HRTEM image in Fig. 3(d) clearly shows that the lattice fringes were also structurally uniform with a spacing of 0.21 nm, which is in good agreement with the d value of the (110) planes of rhombohedral Bi_2_Te_3_. The SAED pattern shown in the inset of Fig. 3(d) was indexed to the [00*l*] zone axis of rhombohedral Bi_2_Te_3_, indicating that this nanoplate was single-crystalline and had a preferential (00*l*) orientation. Notably, weak forbidden reflections were observed, owing to the broken translational symmetry of the Te/Bi antisite defects, which is consistent with previous reports^28,30,31^.

The atomic composition ratios [Te/(Bi + Te)] of the Bi_2_Te_3_ nanoplates prepared at different reaction temperatures are listed in Table 1. The composition ratio of the nanoplates with single nanopores formed at 190 °C was 0.56, which is slightly lower than the stoichiometric proportion of 0.6. At a reaction temperature of 200 °C, the composition ratio of the nanoplates was 0.54. This result gives insight into the behavior of Te/Bi antisite defects, as shown in Fig. 3(d). The composition ratio of the nanoplates with no nanopores was lower than that of the nanoplates with nanopores; thus, a relatively large amount of Te atoms replaced Bi atoms in the nanoplates without nanopores.

**Table 1:** Atomic composition ratio of the Bi_2_Te_3_.

Reaction temperature (°C)	Atomic composition ratio Te/(Bi + Te)
190	0.56
200	0.54

**Figure 4:**
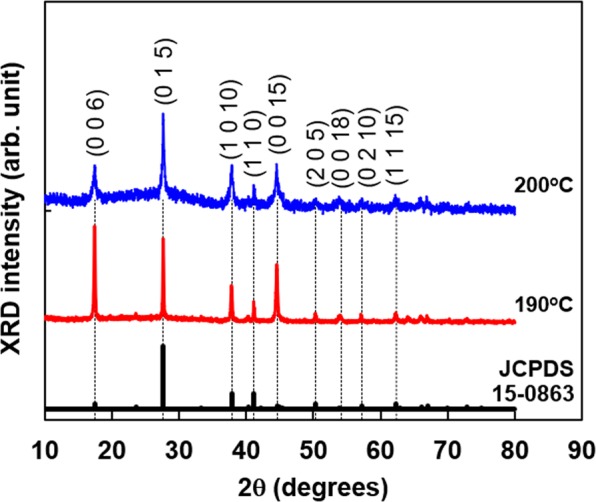
X-ray diffraction patterns of the Bi_2_Te_3_ nanoplates prepared by solvothermal synthesis (**a**) with and (**b**) without nanopores.

The phase purity and crystal structure of the Bi_2_Te_3_ nanoplates were examined by XRD analysis, as shown in Fig. 4. All peaks observed in the XRD patterns of the nanoplates formed at reaction temperatures of 190 °C and 200 °C were indexed to the standard diffraction pattern of Bi_2_Te_3_ (JCPDS 15-0863) although both nanoplates exhibited slight deviations from the exact stoichiometry. The main peaks originated from the (006), (015), (10 10), and (00 15) planes. Therefore, the phase purity and the crystal structure depended only slightly on nanopores in the Bi_2_Te_3_ nanoplates.

**Figure 5:**
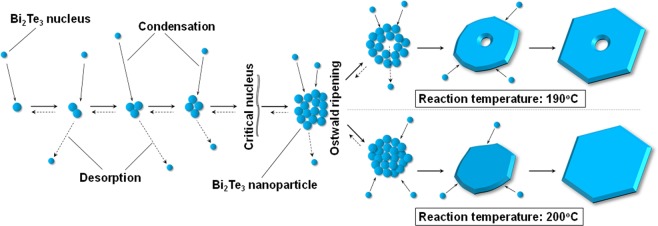
Schematic diagram of the crystal growth of nanoplates with and without single nanopores.

As mentioned above, the single nanopores appeared at the centers of the single-crystalline Bi_2_Te_3_ hexagonal nanoplates at the lower reaction temperature. Here, we propose a growth mechanism and reaction process to account for the structures of these nanoplates. Figure 5 shows a schematic diagram of the crystal growth of the nanoplates with and without single nanopores. When Bi_2_Te_3_ nuclei were generated in the solution, they aggregated. The atomic composition ratios of the nanoplates synthesized at different temperatures deviated slightly from the stoichiometric proportion; however, we consider that this deviation did not affect the aggregation process of the nuclei and Ostwald ripening process described below. When the radius of the aggregated nuclei became larger than the critical nucleus, Bi_2_Te_3_ nanoparticles were generated. In the Bi_2_Te_3_ system, the formation of nanoplates is also attributed to the inherent crystal structure. Because of the high surface energy of the nuclei, the aggregated Bi_2_Te_3_ particles were not in thermodynamic equilibrium and were metastable; Bi_2_Te_3_ nanoplates with a thermodynamic preference for better crystallinity. After formation of Bi_2_Te_3_ nanoplates, the Ostwald ripening process proceeded; however, the process differed at reaction temperatures of 190 °C and 200 °C^32^. At a reaction temperature of 190 °C, Ostwald ripening led to the formation of nanopore structures because of lower crystallinity or less dense particles in the colloidal aggregate, which gradually dissolved, whereas larger, better crystallized or denser particles in the same aggregate continued to grow at the lower reaction temperature. However, at 200 °C, nanopores were not formed in the nanoplates because the particles in the colloidal aggregate did not dissolve owing to the dense structures initially formed at the higher reaction temperature. Both types of Bi_2_Te_3_ nanoplates grew gradually to form deep nanoplates with many crystalline planes from the top to bottom^33,34^. Rhombohedral Bi_2_Te_3_ is built up from many layers extending along the top-to-bottom crystalline planes connected by van der Waals bonds, as shown in Fig. 1.

## Conclusions

We used solvothermal synthesis at different reaction temperatures to prepare Bi_2_Te_3_ hexagonal nanoplates. The structural and crystallographic characteristics of the nanoplates were investigated. The nanoplates synthesized at a lower reaction temperature (190 °C) developed single nanopores (approximately 20 nm in diameter), whereas the nanoplates synthesized at a higher reaction temperature (200 °C) did not have nanopores. Based on the experimental and analytical results, we propose a growth mechanism and reaction process for the nanoplates and the nanopore appearance at different reaction temperatures. We expect that the Bi_2_Te_3_ nanoplates with single nanopores will feature improved thermoelectric performance.

## Supplementary information

12_11_SuppleInfo_ver2
