# Supplementary material for: Growth of single-crystalline Bi2Te3 hexagonal nanoplates with and without single nanopores during temperature-controlled solvothermal synthesis
Source: Sci Rep. 2019 Jul 25;9:10790. doi: 10.1038/s41598-019-47356-5 (PMC6658664; doi:10.1038/s41598-019-47356-5)
Supplement: Supplementary file 1 — 12_11_SuppleInfo_ver2 [file 41598_2019_47356_MOESM1_ESM.docx]

**Growth of single-crystalline Bi_2_Te_3_ hexagonal nanoplates with and without single nanopores during temperature-controlled solvothermal synthesis**

Yuichi Hosokawa ^1^, Koji Tomita ^2^, Masayuki Takashiri ^1,*^

^1^*Department of Materials Science, Tokai University, 4-1-1 Kitakaname, Hiratsuka, Kanagawa 259-1292, Japan*

^2^*Department of Chemistry, Tokai University, 4-1-1 Kitakaname, Hiratsuka, Kanagawa 259-1292, Japan*

**Supplemental information**

In Fig. S1, to clarify whether the generation of nanopores occurred in a limited range or overall, we provide the SEM images of Bi_2_Te_3_ nanoplates at 190°C and 200°C with lower magnification. In the reaction temperature of 190°C, almost all nanoplates had a nanopore in the center (Fig. S1(a)). On the other hand, in the reaction temperature of 200°C (Fig. S1(b)), the nanoplates with a nanopore in the center were not completely seen. Therefore, we conclude that the nanopores in Bi_2_Te_3_ nanoplates were generated due to the differences in the reaction temperature.

**(a)**

**(b)**

Figure S1. Low magnification SEM images of the Bi_2_Te_3_ nanoplates at (a) 190^o^C and (b) 200^o^C.

Figure S2 shows the surface morphologies of Bi_2_Te_3_ nanoplates at 180°C and 230°C using FE-SEM. As a result, single nanopores were obtained in the nanoplates at 180°C (Fig. S2(a)), but not in those formed at 230°C (Fig. S2(b)).

Figure 2S. SEM images of the Bi_2_Te_3_ nanoplates synthesized at (a) 180°C and (b) 230°C.
